# Practical Notes

**Published:** 1909-11-06

**Authors:** 


### PRACTICAL NOTES.

**A New Test for Albumin in Urine.**

Five parts of urine, diluted with 10 or 15 parts of water, is carefully floated on 3 parts of potassium iodide solution and 2 drops of acetic acid, 36 per cent., in a test-tube. In presence of albumin, a white ring is formed at the zone of contact; immediately if as much as 0.01 or 0.02 per cent. is present, and in two minutes with as little as 0.005 per cent.

**Syphilitic Ulceration of the Rectum.**

This is not very painful, and is, therefore, often allowed to continue without treatment for long periods of time. Stricture of the rectum is a consequence of this neglect, and the patient may seek advice for the stricture rather than for the ulceration. In such cases the stricture is narrow, tubular and of considerable length. It is due to cicatrisation of all the coats of the bowel, and is, therefore, hard and dense. In ulcerative colitis, on the other hand, the narrowing of the bowel is confined to the mucous and submucous coats, so that the constriction is more easily dilated.—*Mr. D'Arcy Power.*

